# Aberrant Whole-Brain Transitions and Dynamics of Spontaneous Network Microstates in Mild Traumatic Brain Injury

**DOI:** 10.3389/fncom.2019.00090

**Published:** 2020-01-15

**Authors:** Marios Antonakakis, Stavros I. Dimitriadis, Michalis Zervakis, Andrew C. Papanicolaou, George Zouridakis

**Affiliations:** ^1^Institute for Biomagnetism and Biosignal Analysis, University of Muenster, Muenster, Germany; ^2^Digital Image and Signal Processing Laboratory, School of Electronic and Computer Engineering, Technical University of Crete, Chania, Greece; ^3^Neuroinformatics Group, Cardiff University Brain Research Imaging Center (CUBRIC), School of Psychology, Cardiff University, Cardiff, United Kingdom; ^4^Institute of Psychological Medicine and Clinical Neurosciences, Cardiff University School of Medicine, Cardiff, United Kingdom; ^5^Cardiff University Brain Research Imaging Center (CUBRIC), School of Psychology, Cardiff University, Cardiff, United Kingdom; ^6^School of Psychology, Cardiff University, Cardiff, United Kingdom; ^7^Neuroscience and Mental Health Research Institute, Cardiff University, Cardiff, United Kingdom; ^8^MRC Centre for Neuropsychiatric Genetics and Genomics, School of Medicine, Cardiff University, Cardiff, United Kingdom; ^9^Departments of Pediatrics, and Anatomy and Neurobiology, Neuroscience Institute, University of Tennessee Health Science Center, Le Bonheur Children's Hospital, Memphis, TN, United States; ^10^Biomedical Imaging Lab, Departments of Engineering Technology, Computer Science, Biomedical Engineering, and Electrical and Computer Engineering, University of Houston, Houston, TX, United States

**Keywords:** MEG, mTBI, beamforming, dynamic functional connectivity analysis, network microstates, symbolic dynamics, chronnectomics, connectomic biomarkers

## Abstract

Dynamic Functional Connectivity (DFC) analysis is a promising approach for the characterization of brain electrophysiological activity. In this study, we investigated abnormal alterations due to mild Traumatic Brain Injury (mTBI) using DFC of the source reconstructed magnetoencephalographic (MEG) resting-state recordings. Brain activity in several well-known frequency bands was first reconstructed using beamforming of the MEG data to determine ninety anatomical brain regions of interest. A DFC graph was formulated using the imaginary part of phase-locking values, which were obtained from 30 mTBI patients and 50 healthy controls (HC). Subsequently, we estimated normalized Laplacian transformations of individual, statistically and topologically filtered quasi-static graphs. The corresponding eigenvalues of each node synchronization were then computed and through the neural-gas algorithm, we quantized the evolution of the eigenvalues resulting in distinct network microstates (NMstates). The discrimination level between the two groups was assessed using an iterative cross-validation classification scheme with features either the NMstates in each frequency band, or the combination of the so-called chronnectomics (flexibility index, occupancy time of NMstate, and Dwell time) with the complexity index over the evolution of the NMstates across all frequency bands. Classification performance based on chronnectomics showed 80% accuracy, 99% sensitivity, and 49% specificity. However, performance was much higher (accuracy: 91–97%, sensitivity: 100%, and specificity: 77–93%) when focusing on the microstates. Exploring the mean node degree within and between brain anatomical networks (default mode network, frontoparietal, occipital, cingulo-opercular, and sensorimotor), a reduced pattern occurred from lower to higher frequency bands, with statistically significant stronger degrees for the HC than the mTBI group. A higher entropic profile on the temporal evolution of the modularity index was observed for both NMstates for the mTBI group across frequencies. A significant difference in the flexibility index was observed between the two groups for the β frequency band. The latter finding may support a central role of the thalamus impairment in mTBI. The current study considers a complete set of frequency-dependent connectomic markers of mTBI-caused alterations in brain connectivity that potentially could serve as markers to assess the return of an injured subject back to normality.

## Introduction

Mild traumatic brain injury (mTBI) accounts for ~90% of all brain injuries (Len and Neary, 2011), establishing it as a major cause of brain insult (Huang et al., 2014). A considerable part of mTBI patients develops persistent cognitive deficits (van der Naalt et al., 1999; Vanderploeg et al., 2005), and post-concussion symptoms can cause irremediable problems in ~20% of the patients (Bharath et al., 2016) several months after the first injury (Huang et al., 2014). The main characteristics of those symptoms are often physical, emotional, cognitive, and sleep disturbances that may need several months to improve (Huang et al., 2014). In many neuropsychological studies (Huang et al., 2014; Pang et al., 2016), reduced cognitive efficiency in several brain functions has been reported, especially in tests measuring processing speed, executive function, attention, memory, and connectivity, in mTBI patients with persistent symptoms. Handling of mTBI patients is not a trivial task as oftentimes mTBI affects severely brain functionality (Vanderploeg et al., 2005; De Monte et al., 2006). In the present study, we aim to reveal abnormal alterations due to mTBI using magnetoencephalographic resting-state data and dynamic functional connectivity (DFC) patterns in source space.

Conventional structural neuroimaging, such as computed tomography (CT) and acute magnetic resonance imaging (MRI), as well as functional MRI (fMRI) usually offer low sensitivity for detecting physiological alterations caused by mTBI (Kirkwood et al., 2006). A recent study (Vergara et al., 2018) revealed high classification levels of mTBI subjects exploiting time resolved connectivity profiles but with temporal limitations due to the use of fMRI. Magnetoencephalography (MEG) is a non-invasive functional imaging modality that detects activity from the synchronous oscillations of neurons' membranes in the gray matter. Thus, MEG incorporates high sensitivity by keeping the environmental noise to a low level, and includes low-resolution spatial details and high temporal accuracy (Leahy et al., 1998). In this study, we combined for the first time the reconstructed source MEG activity with the notion of functional connectivity (FC) for the characterization of mTBI over time. FC is crucial for the characterization of most brain disorders (Eierud et al., 2014; Baillet, 2017). The term FC was introduced when the human brain was first modeled as a neurophysiological network with functional communication among several anatomical areas. These distinct networks can exist in a range of spatiotemporal scales with spatial diversity and temporal variability. Spatially, these networks can vary between microscopic neuronal aggregates and large-scale interconnected brain areas (Eierud et al., 2014).

Several studies, including ours, have recently investigated the development of robust biomarkers for detecting mTBI using MEG or fMRI and under the notion of FC (Huang et al., 2009; Castellanos et al., 2010; see reviews by Jeter et al., 2013; and Eierud et al., 2014; Da Costa et al., 2015; Dunkley et al., 2015; Vakorin et al., 2016). More recently, Dunkley et al. (2018) investigated the impact of the injury on intrinsic connectivity networks and showed increased coupling in the default mode network of mTBI patients. Dimitriadis et al. (2015), using phase-coupling, quantified intra-frequency couplings at the sensor level and found significantly different patterns that were seen mostly in the delta band, whereas Alhourani et al. (2016) used the same metric at the source level and showed reduced local efficiency in different brain regions in mTBI patients. In a series of follow-up studies, we (Antonakakis et al., 2016, 2017a) showed less dense connectivity networks in mTBI patients, which was in line with the findings of other groups (Rapp et al., 2015), as well as higher synchronization among mTBI rich-club hubs (Antonakakis et al., 2017b). More recently, Li et al. (2018) revealed a denser causality network for mTBI patients, whereas Kaltiainen et al. (2018) showed that aberrant theta-band activity could provide an early objective sign of brain abnormality after mTBI.

MEG-based FC is an emerging procedure in the development of reliable mTBI biomarkers using resting-state networks (RSNs), not only at the sensor level, but also at the source level, since source level RSNs have been successfully estimated in the past few years (Brookes et al., 2011a,b; Hipp et al., 2012; Luckhoo et al., 2012; Hall et al., 2013; Wens et al., 2015). MEG-based RSNs form a promising approach for detecting several other brain functional abnormalities, involving dyslexia (Dimitriadis et al., 2016), mild cognitive impairment (Maestú et al., 2015), and multiple sclerosis (Tewarie et al., 2015). However, the use of RSNs as short-lived transient brain states (van Dijk et al., 2010) varies significantly across different studies, ranging from estimating the MEG frequency spectrum (Vidaurre et al., 2016) to band-specific amplitude envelopes of reconstructed MEG sources (Baker et al., 2014; O'Neill et al., 2015; Vidaurre et al., 2016). A recent neuroimaging index, called chronnectomics (Allen et al., 2012; Calhoun and Adali, 2016), was proposed in fMRI studies to express the synergy between time-varying FC and the evolution of distinct spatiotemporal alternations among various brain states.

In this study, we investigated whole-brain dynamic FC (DFC) derived from MEG resting-state data from 80 subjects (50 HC and 30 mTBI). To our knowledge, this is the first study that utilizes DFC on reconstructed MEG source activity for the investigation of mTBI. Similar methodological aspects have been adopted in other recent studies with normal controls and mild cognitive impairment subjects (Dimitriadis et al., 2018a).

Within brain interactions were modeled using beamformed MEG source activation and DFC among ninety atlas-based brain areas using a template MRI. Each quasi-static FC was determined by the imaginary part of the phase locking value (iPLV) (Dimitriadis et al., 2018a; Palva et al., 2018), and it was filtered statistically and topologically (Dimitriadis et al., 2017) to reduce spurious connections. Subsequently, we coded the estimated time-varying network activity into prototypical network microstates or NMstates (Dimitriadis et al., 2013, 2018a). Through this approach, we derived symbolic-form time series for which the chronnectomic behavior was modeled based on the metric transition rate of NMstates, fractional occupancy of each NMstate, Dwell time, and complexity index (Dimitriadis et al., 2018b). Finally, we assessed DFC differentiations of mTBI subjects using statistical inference and classification.

The rest of the paper is organized as follows: section Materials and Methods describes in detail the study participants, data acquisition, preprocessing steps, the methodological approach, and the resulting chronnectomics. Section Results is devoted to the description of findings based on the frequency-dependent prototypical network NMstates and the extracted chronnectomics. Section Discussion includes a discussion on the analysis results in the context of the current literature, and its potential impact on the field. Finally, section Discussion summarizes the conclusions of the study and presents possible future extensions.

## Materials and Methods

### Participants and Recordings

The current study included data from 50 right-handed healthy controls (HC) (29.25 ± 9.1 years of age) and 30 right-handed mTBI patients (29.33 ± 9.2 years of age). The participants gave written informed consent to the study. All clinical information, including the selection criteria, was reviewed and provided by board certified clinicians. Table 1 summarizes patient demographics. Controls were recruited from a normative data repository at UTHSC-Houston and were particularly selected so that they were age-matched with the mTBI group. The selected healthy control subjects had no previous head injuries, extensive dental work, substance abuse, history of neurologic or psychiatric disorder, or implants incompatible with MEG. Prior to the present study, the research protocol received institutional approval. The mTBI patients were recruited from three trauma centers in the greater Houston metropolitan area that participated in a larger study (Zouridakis et al., 2012). Further details can be found elsewhere (Zouridakis et al., 2012; Dimitriadis et al., 2015; Antonakakis et al., 2016). Characterization of mTBI patients was based on the guidelines of the American Congress of Rehabilitation Medicine (Kay et al., 1993) and the Department of Defense (Assistant Secretary, 2007). Institutional Review Board (IRB) approval for the project was obtained at the participating institutions and the Human Research Protection Official's review of research protocols for the Department of Defense. All procedures were fully compliant with the Health Insurance Portability and Accountability Act (HIPAA).

**Table 1:** Patient demographics for the mTBI group.

**Age at injury (min—max)**	**Males (females)**	**Auto pedestrian—frontal (# subjects)**	**Auto pedestrian—frontal—type (# subjects)**	**Auto pedestrian—frontal—location (# subjects)**
(19–25)	7 (5)	Assault (2), Motor Vehicle (5), Sports-related (2), Auto Pedestrian (1), ATV (1), Assault (1)	Contusion (4), Bruising (3), Laceration—no sutures (1), Tenderness (2), Laceration—with sutures (2)	Head (10), Head/Face (2)
(25–40)	8 (2)	Fall (1), Auto Pedestrian (2), Fall Moving Object (1), Assault (1), Motor Vehicle (1), Fall Raised Surface (2), Assault (1), Blow to Head (1)	Abrasion (3), Bruising (1), Tenderness (2), Contusion (3), Laceration—no sutures (1)	Head (9), Head/Face (1)
(40–50)	3 (5)	Motor Vehicle (3), Assault (1), Fall Standing (2), Motorcycle (1), Fall Moving Object (1)	Abrasion (1), Bruising (1), Tenderness (4), Laceration—no sutures (1), Tenderness (1), Contusion (1), Laceration—with sutures (1)	Head (8)

*The first column shows the age at injury as a range. The second column presents the number of genders separately. The rest of the columns indicate the type of injury while the number of patients is noticed within the parenthesis*.

Spontaneous MEG activity was acquired with a whole-head Magnes WH3600 system of 248 axial gradiometers (4D Neuroimaging Inc., San Diego, CA) for 10 min at a sampling rate of 1,017.25 Hz. An online bandpass filter between 0.1 and 200 Hz was applied to reduce noise effects. No independent ocular or cardiac activity was recorded. Subjects were in a supine position with eyes closed during data acquisition. After excluding activity contaminated with artifacts (Dimitriadis et al., 2015) and conversion from axial gradiometer recordings to planar gradiometer field approximations in FieldTrip (Oostenveld et al., 2011), ~5 min of clean data were used for further analysis.

### MEG Pre-processing

Artifact reduction in the MEG recordings was accomplished with an automated detection and elimination procedure described in detail elsewhere (Antonakakis et al., 2017a) that was based on the FieldTrip software (Oostenveld et al., 2011) implemented in MATLAB (The MathWorks, Inc., Natick, MA, USA). In brief, noisy activity was attenuated using the following steps: (1) correction of bad MEG channel activity by applying interpolation techniques, (2) elimination of frequencies outside the range 0.1–100 Hz using digital filtering, (3) elimination of the power line noise at 60 Hz with a notch filter, and (4) detection and elimination of electrophysiological (ocular and cardiac) artifacts by first decomposing the MEG signals into statistically independent components (Delorme and Makeig, 2004) and then applying combined fixed thresholds on the statistical values of kurtosis, skewness, and Rényi entropy as described in detail elsewhere (Antonakakis et al., 2017a).

### Source Analysis

Atlas-based beamforming was used for reconstructing source activity from MEG measurements. The investigated frequency bands included δ (0.5–4 Hz), θ (4–8 Hz), α_low_ (8–10 Hz), α_high_ (10–13 Hz), β (13–30 Hz), γ_low_ (30–55 Hz), and γ_high_ (55–90 Hz). First, the MEG sensor locations of each subject were realigned with a standard T1-weighted MRI template of 2 mm resolution provided by SPM8 (Weiskopf et al., 2011). The division of the MRI anatomical areas into 90 brain regions of interest (ROIs) was performed based on the Automated Anatomical Labeling (AAL) atlas (Hillebrand and Barnes, 2002; Tzourio-Mazoyer et al., 2002; Hillebrand et al., 2016; Hunt et al., 2016). We employed a spherical head model (Nolte et al., 2004) that included 5,061 sources (6 mm resolution) and covered the entire brain tissue. Frequency-depended MEG source activity was reconstructed using the linearly constrained minimum norm variance (LCMV) algorithm in FieldTrip (Oostenveld et al., 2011).

**Figure 1:**
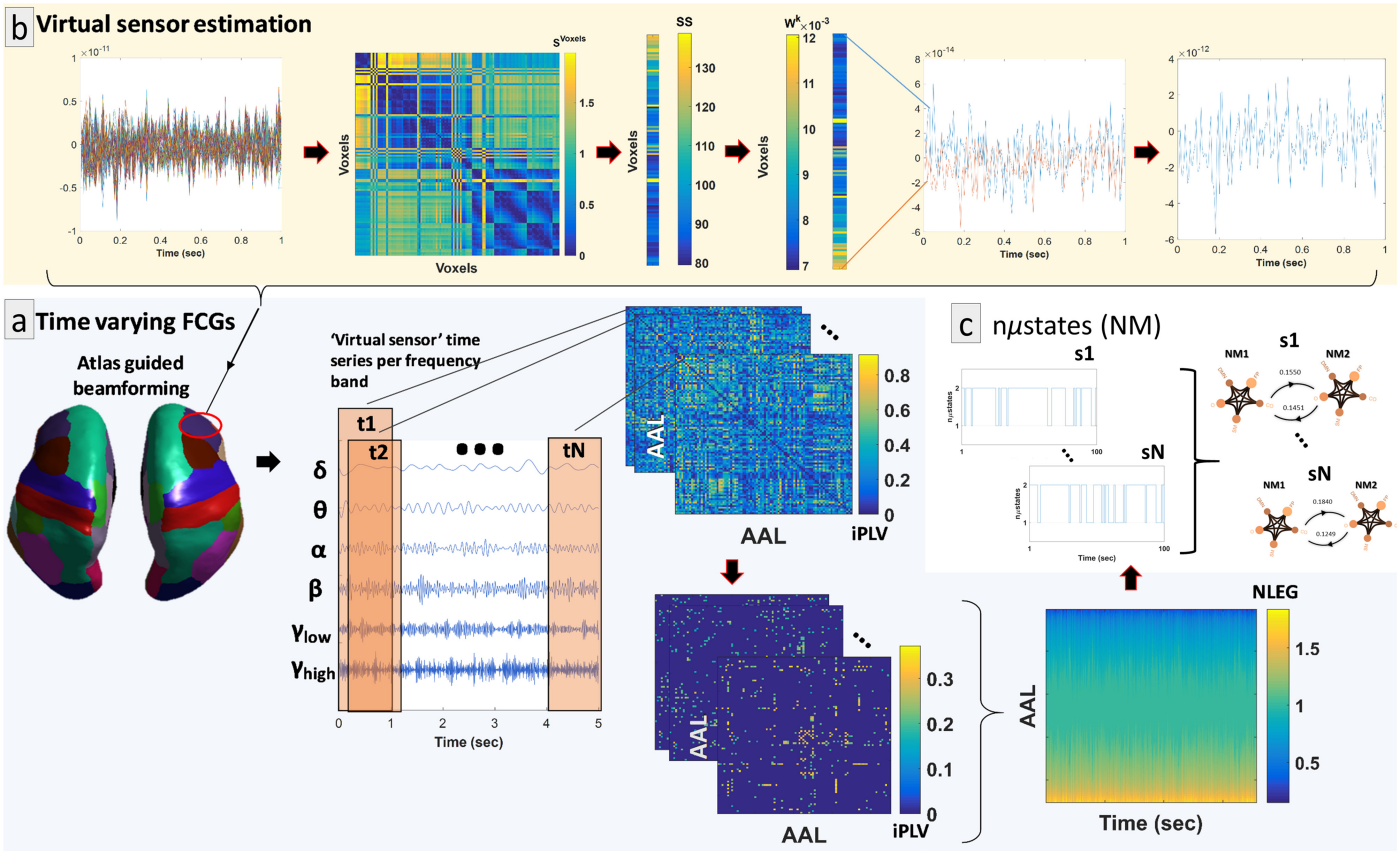
Overview of the proposed source analysis procedure. **(a)** Schematic representation of the basic steps for estimating time-varying (or dynamic) functional connectivity graphs (DFCGs) for every subject. From left to right, cortical atlas parcellation of the brain regions of interest (ROI); the represented time-series of each ROI filtered for every brain rhythm; topologies of snapshots of the imaginary part of the phase locking value (iPLV) DFGC from the first two (t1 and t2) and the last (tN) temporal segments; statistically and topologically filtering of those topologies; dynamic evolution of the eigenvalues of the normalized Laplacian matrices (NLEG). **(b)** Steps for determining the representative virtual sensor for each ROI. (Left to right) An example of the time series obtained from voxels correspond to right middle frontal gyrus. Distance correlation matrix (S^Voxels^) derived by pair-wise estimations across the voxel-based time series. Summation of the S^Voxel^ columns produced vector SS. Normalization of vector SS resulted in a new vector W^k^ whose components sum to 1. Multiplication of every voxel time series with the related weight from the W^k^. This example shows multiplication only for the first and last voxel time series. The weighted versions of the all-time series were summed for the final estimated virtual sensor for each ROI. **(c)** Short representation of the NMstates procedure showing a sample of the symbolic time series and the corresponding prototypical NMstates in a circular visualization with nodes the degree of basic anatomical networks (DMN, default mode network; FP, frontoparietal; OCC, occipital; CO, cingulo-percular; SM, sensory motor) and edges the strength of the iPLV. A schematic example from the 1st up to the Nth are presented.

By adopting similar methodological modules as in a recent study (Dimitriadis et al., 2018a), we determined a representative source signal for every ROI. The entire procedure is illustrated in Figure 1b using an example of time series data obtained from a specific brain region of interest (ROI) (right hemisphere—middle frontal gyrus). In more detail, the contribution of every MEG sensor was weighted by the LCMV beamformer for the reconstruction of a voxel-based time series for the entire predefined grid. The projection of the MEG sensor activity to the source point was performed by means of spatial filters. Each atlas-based ROI contains a different number of voxels. Subsequently, we estimated ROI representative virtual sensors by interpolating functional activity from the voxel time series of individual ROIs (Dimitriadis et al., 2018a). Within each atlas ROI, we estimated the correlation between all possible pairs of source time series in order to map all voxel temporal associations in a common graph (Figure 1b, second image from the left). The next step was the calculation of the node-strength of each voxel within the ROI. The strength was determined by summing the connectivity values between a specific node-voxel with the rest of the node-voxels within the same ROI. Then, we normalized the strength values to weights with sum equal to 1 within the ROI. The procedure ended with the estimation of a representative time series for each ROI by summing up across the voxel time series multiplied with their respective weights. This procedure is depicted in Figure 1b (upper row, left to right).

### Dynamic Functional Connectivity Graphs

In the present study, DFC graphs (DFCG) were calculated separately for each frequency band described previously. The imaginary part of the Phase Locking Value (Dimitriadis et al., 2018a; Palva et al., 2018) or iPLV was used as an FC estimator, a metric that has shown good sensitivity to non-zero-phase lags and tolerability to instantaneous self-interactions from volume conductance (Palva et al., 2018). Given a pair of two phase signals, φ_*x*_(*t*) and φ_*y*_(*t*), derived from the application of the Hilbert transformation to the original signals *x*(*t*) and *y*(*t*), the iPLV is described as follows:

(1) $$\begin{array}{r} {\text{iPLV}}_{xy}=\left|im\left(\underset{t}{\sum }e^{i\left(\varphi_{x}\left(t\right)-\varphi_{y}\left(t\right)\right)}\right)/N\right| \end{array}$$

where *N* is the number of samples and |.| denotes the absolute value operator.

We applied this metric in a dynamic manner for understanding better the time-varying changes of phase-to-phase interactions. This was achieved by computing the iPLV within a series of shifted and overlapping windows, spanning the entire 5-min continuous ROI time series (Figure 1a, middle column). The window length of each temporal segment (or timestamp) was set to 2 s with an overlap of 10% for each frequency band. The resulting number of timestamps was equal to 1,785 per frequency band and subject.

#### DFCG Filtering

##### Statistical filtering of the DFCGs

A surrogate analysis was performed to evaluate the non-spurious iPLV connections on each sliding window (i.e., timestamp) for every frequency band. The null hypothesis H_0_ examines whether the given iPLV coupling belongs to the empirical distribution estimated by the surrogates. We generated ten thousand surrogate time series to test this hypothesis by selecting a random cut-point in the middle of the time series and changing thereafter the order of the two reproduced temporal segments. We repeated the same procedure for each of the 90-source virtual time series (Aru et al., 2015). This procedure ensured similar statistical properties for both original and surrogate iPLV (iPLV_s_). After estimating the empirical distribution, a statistical level of significance was determined for every iPLV by estimating the amount of iPLV_s_ that was higher than the original iPLV. The *p*-value was set to 0.05. A further condition was applied for assessing multiple comparisons within each quasi-static FCG (a 90 × 90 matrix with tabulated *p*-values) with the expected fraction of false positives being at the level of 0.01 (Benjamini and Hochberg, 1995). The non-significant values were set to zero, and the final DFCG had a 3D dimension of 1,785 (segments) × 90 (sources) × 90 (sources) per subject and frequency band.

##### Topological filtering of the DFCGs

In addition to statistical filtering, a data-based topological connection-cutting scheme was applied, based on a recently suggested procedure (Dimitriadis et al., 2017). The so-called Orthogonal Minimal Spanning Trees (OMST^1^ was performed to uncover the entire structure of the most dominant paths within every quasi-static FCG (Figure 1a, middle column). The OMST procedure initially emerged from the notion that a fully connected FCG can be reduced to an acyclic FCG or MST of minimum cost from the root node to leaf node without changing the ordered strength of connections (Dimitriadis et al., 2017). Subsequently, the global efficiency of the specific MST was optimized, preserving the same total cost among the connections (Dimitriadis et al., 2017). The resulting DFC profiles were 3D arrays of size 1,785 (timestamps) × 90 (sources) × 90 (sources) for every subject and frequency band.

#### Symbolization of the DFCG

In this subsection, we briefly introduce our methodological procedure and necessary notations which have been presented in great detail in previous studies (Dimitriadis et al., 2018a). The dynamic connectivity patterns (DFCG) can be transformed into prototypical network microstates (NMstates) based on a vector quantization procedure for effective DFCG modeling (see section Methods in Supplementary Material). The input of this procedure was a 2D matrix *V*. The steps for calculating this matrix are summarized in the following paragraph.

We first calculated the normalized Laplacian matrix (Chung and Graham, 1997) for every quasi-static FCG based on the equation,

(2) $$\begin{array}{r} L=I-D^{-\frac{1}{2}}\cdot G\cdot D^{-\frac{1}{2}} \end{array}$$

where *I* denotes the identity matrix, *D* is the degree matrix and G is the quasi-static FCG [90 ROI × 90 ROI] from every subject and frequency band. Then, eigenvalue analysis (*N*_eigenvalues_ = 90) on the normalized Laplacian transformations was employed to reveal the synchronization level of the original FCGs (Figure 1a, right column). In this form, the richness of information existing in the DFC profiles was represented by a decomposition matrix U that described the assignment of input *V* to code vectors. In the current work, we used the statistically and topologically filtered DFCGs in their inherent format, i.e., as 3D tensors (the third dimension was time). This step derived the 2D matrix *V* with the first dimension denoting the number of decomposed eigenvalues (90) and the second the number of timestamps (1,785).

Subsequently, we followed a vector quantization process (Dimitriadis et al., 2013) for modeling individual DFC profiles as NMstates (or NM_i_ for i = 1, …, k) of a small number k symbols (Figure 1c). An approximation for the vector quantization process was obtained by the neural-gas (NG) algorithm (Martinetz et al., 1993). The NG model implements an artificial neural network that converges efficiently to a small number *k* of codebook vectors with negligible loss of information (see details of the algorithm in section Methods in Supplementary Material). A stochastic gradient descent procedure with a softmax adaptation rule minimized the distortion error *DE* between the original data vector (*v*(*t*) ∈ *V*) and the reconstructed *v*_*rec*_(*t*) as follows:

(3) $$\begin{array}{r} DE=\frac{\sum_{t=1}^{N_{total}}∥v\left(t\right)-v_{rec}\left(t\right){∥}^{2}}{\sum_{t=1}^{N_{total}}∥v\left(t\right)-\bar{v}{∥}^{2}}\\ for\;t=1,\ldots ,T\;and\;\bar{v}=\frac{1}{N_{total}}\overset{N_{total}}{\underset{t=1}{\sum }}v\left(t\right) \end{array}$$

where *T* = 1,785 timestamps. The smaller the *DE*, the better the encoding. This index gets smaller with the increase of k. The total number of the representative symbols was fixed at *k* = 2. For this k, the *DE* between the original FC^L−EIGEN^ time series (L: Laplacian – Eigen: Eigenanalysis) and the reconstructed ${\text{FC}}_{\text{rec}}^{\text{L}-\text{EIGEN}}$ time series (based on the symbolic time series) was <2% for every subject and frequency band. The estimated normalized Laplacian-based symbolic times series that preserved the information of NMstates are indicated hereafter as STS^L−EIGEN^. An STS^L−EIGEN^ can be viewed as a first-order Markovian chain that describes the temporal evolution of NMstates for every subject.

After computing individual NMstates in every single subject, we estimated the cosine similarity between the *k* = 2 NMstates of every single subject, constructing a similarity matrix of size [2 × subjects] × [2 × subjects] independently for each group. Afterwards, we applied K-means clustering in order to organize NMstates within the subjects of every cohort. Based on the silhouette index, the number of clusters was optimal for *k* = 2. At group level, we estimated the cosine similarity between the [2 NMstates × HC] and [2 NMstates × mTBI] and, based on the highest cosine value, we aligned NMstates between the 2 groups.

#### Network Metrics Derived From NMstates and Brain Subnetworks

In every node of the statistically and topologically filtered quasi-static FCG, we calculated the mean degree and strength across the NMstates by employing subject-specific STS. In particular, the degree was defined as the total number of connections in every node resulting in a vector of 90 values per quasi-static graph, brain rhythm, and subject [90 (ROIs) × 1,785 (temporal segments) × 6 (frequencies) × 80 (HC + mTBI subjects)]. Moreover, to better understand the network connectivity in the whole brain, we estimated the mean degree within and between the following five brain anatomical subnetworks (BAN) in pairs, the default mode network (DMN), the sensorimotor (SM), the frontoparietal (FP), the occipital (OCC), and the cingulo-opercular (CO) (these abbreviations were used to present interactions among these brain networks). We followed the same analysis for the estimation of mean functional strength within and between the five brain subnetworks.

First, we summarized the mean degree in every brain region, within and between five subnetworks for every NMstate across frequency bands in both subject groups, for every brain region. A statistical assessment on the mean degree was followed separately for each NMstate, across all six frequency bands and in every brain region, within the five brain subnetworks and among them (overall ten combinations). Mean degree was estimated independently for each subject.

We then adopted a statistical procedure to estimate the significance level for each brain region within and between the five brain subnetworks per frequency band and NMstate. The procedure included a normality control based on the Kolmogorov-Smirnov test and, depending on the outcome, the use of either the parametric pair-wise sample *t-test* or the non-parametric pair-wise Mann-Whitney u-test (Antonakakis et al., 2016). The significance threshold of the *p*-value was set to 95% (*p* < 0.05). By adopting the False Discovery Rate (FDR) adjustment (Benjamini and Hochberg, 1995), we corrected the resulting *p*-values for multiple comparisons.

#### Temporal Evolution of Modularity Organization of NMstates

To track fluctuations in topological mapping (TM) over time, we followed a novel analysis scheme that did not require labeling of each node into a pre-defined topological mapping class (Shine et al., 2016). For every frequency band and NMstate, we first applied the modularity algorithm to partition each brain network into a number of classes and then we computed the modularity index *Q* that shows the quality of the partition, the module degree Z-scored, *W*_τ_ and the participation coefficient, *B*_τ_, averaged across the network. A joint histogram of within-module (module degree z-scored, *W*_τ_) and between-module (participation coefficient, *B*_τ_) network metrics, named as *topological mapping profile* (Shine et al., 2016), was produced. This profile was calculated for each NMstate for assessing whether the resting brain representation fluctuates over time between the two network microstates. The first step on calculating the TM was to define the modularity index, which statistic quantifies the degree to which the network may be subdivided into such delineated groups (Rubinov and Sporns, 2010). A fine-tuning algorithm from the Brain Connectivity Toolbox^2^ was used to estimate this statistic for every timestamp, frequency band, and subject. Based on the same toolbox, we then estimated the within-module connectivity by employing the time-resolved module-degree z-score (*W*_τ_; within module strength) for each region *r* in our analysis (Shine et al., 2016).

(4) $$\begin{array}{r} W_{r\tau }=\frac{\kappa_{r\tau }-\kappa_{s_{r\tau }}}{\sigma_{\kappa_{s_{r\tau }}}} \end{array}$$

where κ_*s*_*rτ*__ is the strength of connections from region *r* to other regions in its module *s*_*r*_ at time τ, κ_*s*_*rτ*__ is the average of κ over all the regions in *s*_*r*_ at time τ, and σ_κ__*s*__*rτ*___is the standard deviation of κ in *s*_*r*_ at time τ.

The participation coefficient, *B*_τ_, is a metric for the quantification of the extent to which a region connects across all modules (i.e., between-module strength). The *B*_τ_ was calculated within each timestamp *B*_τ_ for each brain region based on the following equation:

(5) $$\begin{array}{r} B_{r\tau }=1-\overset{n_{M}}{\underset{s=1}{\sum }}{\left(\frac{\kappa_{rs\tau }}{\kappa_{r\tau }}\right)}^{2} \end{array}$$

where κ_*rsτ*_ is the strength of the positive connections of region *r* to regions in module s at time τ, and κ_*rτ*_ is the sum of strengths of all positive connections of region *r* at time τ. The value of *B*_*rτ*_ on a region *r* is close to 1 or 0 if its connections are uniformly distributed among all the modules or its connections are only within its own module, respectively.

In the final comparison stage we followed two directions, specifically (1) we estimated the relative difference of topological mapping profiles between the two groups for each NMstate and (2) we compared the two NMstates in terms of *Q*, *B*_τ_ and *W*_τ_ for every frequency band and group. For the latter comparison, the statistical analysis scheme was the same as described in subsection Network Metrics Derived From NMstates and Brain Subnetworks, where this time the inputs were grouped by NMstate, not by group (HC or mTBI).

#### Chronnectomics: Characterization of Temporal Dynamics of NMstates

We calculated DFC metrics based on the so-called *chronnectomic* features, which were estimated from the STS^L−EIGEN^ that expressed changes among the NMstates (Dimitriadis et al., 2018b). The first metric was the flexibility index (FI) that expressed the transition rate among the NMstates and was estimated from the following equation,

(6) $$\begin{array}{r} FI=\frac{number\;of\;transitions}{slides-1} \end{array}$$

where *slides* denotes the number of timestamps or temporal segments. FI yields higher values for increased numbers of brain “hops” between the NMstates. The next metric was the Occupancy Time (OT) that accounts for the percentage of occurrence of an NMstate across the experimental time and is computed as

(7) $$\begin{array}{r} OT\left(k\right)=\frac{frequency\;of\;Occurance}{slides} \end{array}$$

where *slides* denote the number of timestamps and *k* denotes the NMstates. Another metric was the complexity index (CI) of an STS^L−EIGEN^, which was estimated as

(8) $$\begin{array}{r} CI\left(STS^{L-EIGEN}\right)=\overset{n}{\underset{l=1}{\sum }}c^{l}\left(STS^{L-EIGEN}\right) \end{array}$$

where *c*^*l*^(*STS*^*L*−*EIGEN*^) denotes the number of distinct substrings of *STS*^*L*−*EIGEN*^ of length *l* (Dimitriadis et al., 2013, 2018b; Antonakakis et al., 2016). The parameter *l* was set to 10 for all frequency bands and subjects. An additional metric was the Dwell Time (DT), which accounts for the time that the brain spends within a particular NMstate before it transitions to another state. In contrast to the OT chronnectomic, DT is the amount of consecutive periods that the brain sticks to a particular state, whereas OT measures the summation of time that the brain spends on a brain state. The last metric used was the Transition Probability Matrix, which accounts for the pairwise transitions of brain states over a common codebook, scanning the Markovian chain from left to right. For example, if the following STS describes the temporal evolution of three brain states [1 2 2 1 2], then the pairwise transitions (PT) are equal to: PT_12_ = 2/4 = 0.5 and PT_21_ = 1/4 = 0.25. The size of the pairwise transition matrix is equal to (the number of brain states) × (the number of brain states).

We assessed the level of significance of the aforementioned chronnectomic features by adopting a surrogate data analysis. We shuffled 1,000 times the subject-specific STS^L−EIGEN^ resulting in 1,000 surrogate chronnectomics estimates. Then, we assigned a *p*-value to every subject-specific chronnectomic metric by comparing the original value with the 1,000 surrogate values. Finally, we analyzed the subject-specific chronnectomic features that were statistically significant (*p* < 0.01) only at group-level. For pairwise transitions, we created a transition matrix by assigning a *p*-value to each pairwise transition. Subsequently, we controlled for multiple comparisons using FDR, separately in each frequency band.

#### Classification and Statistical Assessments

We followed an iterative 10-fold cross-validation procedure to assess the performance of NMstates in predicting the class label (HC or mTBI) of the test subjects. The 10-fold cross-validation was repeated one thousand times and, in each iteration, cross-validation was repeated twice, separately for each NMstate (vector of 90 eigenvalues) from the STS^L−EIGEN^ time series. To demonstrate the efficiency of our approach, we adopted the simple k-Nearest Neighbor or k-NN classifier (Horn and Mathias, 1990) with *k* = 10. In every fold, we trained the k-NN classifier with the specific training dataset ([no of train subjects × 90 eigenvalues], where the 90 eigenvalues expressed the NMstates) that included data from both groups. We tested its performance on the testing dataset ([no of test subjects × 90 eigenvalues]). In addition to the previous classification scheme, we further tested the discrimination ability of the chronnectomics in predicting the class label of the test subjects across the frequency bands. In this approach, a rank-feature procedure was used for the selection of the ten most dominant chronnectomics in every fold. For both schemes, we quantified classification performance based on index accuracy (percent of the discrimination level), sensitivity (the portion of the actual mTBI labels), and specificity (the portion of the actual HC labels).

Statistical analysis of the feature vectors employed for classification was used to further confirm the discrimination level between the two groups. The null hypothesis H_0_ tested whether the groups contain an equal mean value per feature vector. We examined the H_0_ using either a parametric pair-wise sample *t*-test or a non-parametric pair-wise u-test, based on the outcome of a normality test (Kolmogorov-Smirnov test) on the input data (the statistical steps were the same as the ones described in section Network Metrics Derived From NMstates and Brain Subnetworks). The significance threshold of the *p*-value was set to 95% (*p* < 0.05), corrected by applying the FDR adjustment.

## Results

### From Multichannel Recordings to a Restricted Repertoire of Quasi-Static NMstates

**Figure 2:**
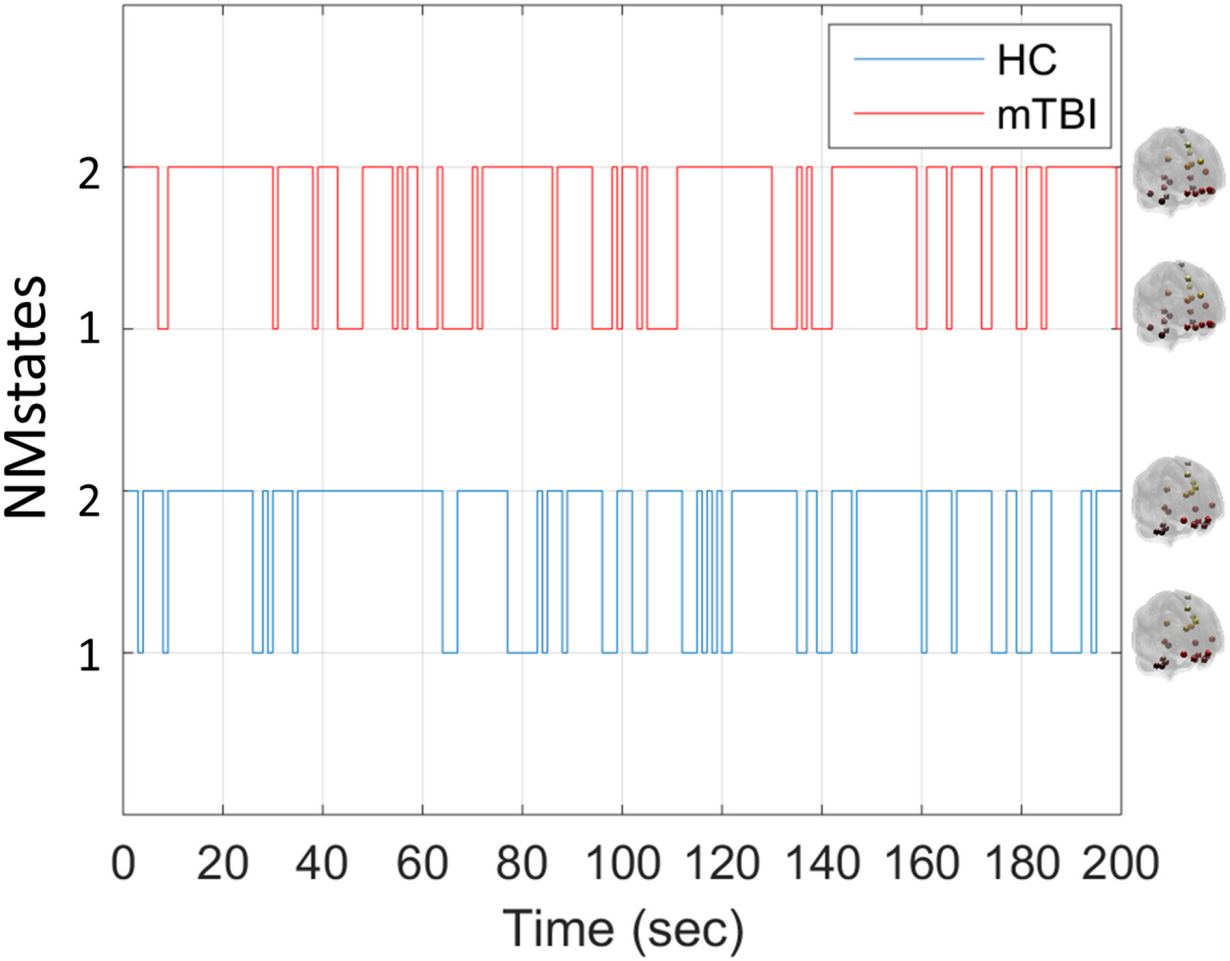
Example of temporal evolution of NMstates for representative HC and mTBI subjects in delta frequency band. Sample symbolic time series for the delta frequency for the HC and mTBI group in order to show how a symbolic time series progresses on time. The brain topologies represent the mean degree of every node that constitutes the default mode network for each NMstate.

Our analytical pipeline revealed unique functional connectivity patterns (NMstates) of phase-synchronized activity that can be considered as discrete brain states: the human brain switches among characteristic NMstates whose temporal evolution can convey important information. The error between the original FC time series and the reconstructed FC time series was <2% for every subject and frequency band. Our analyses thus showed that two NMstates could describe the temporal evolution of eigenvalues in every subject of both groups and across all frequency bands studied. Figure 2 shows two examples of state transitions of NMstates, for a control (HC) and an mTBI subject, using activity in the δ frequency band. Modeling the temporal evolution of eigenvalues results in a symbolic time series, indicated by STS^L−EIGEN^ (Figure 2) that preserves the temporal information of the characteristic NMstate at every temporal segment. In addition to the representative STS^L−EIGEN^, we considered NMstates as brain topologies with nodes five characteristic brain subnetworks (DMN, SM, FP, OCC, and CO), and as connections the mean degree between brain areas located within those subnetworks.

**Figure 3:**
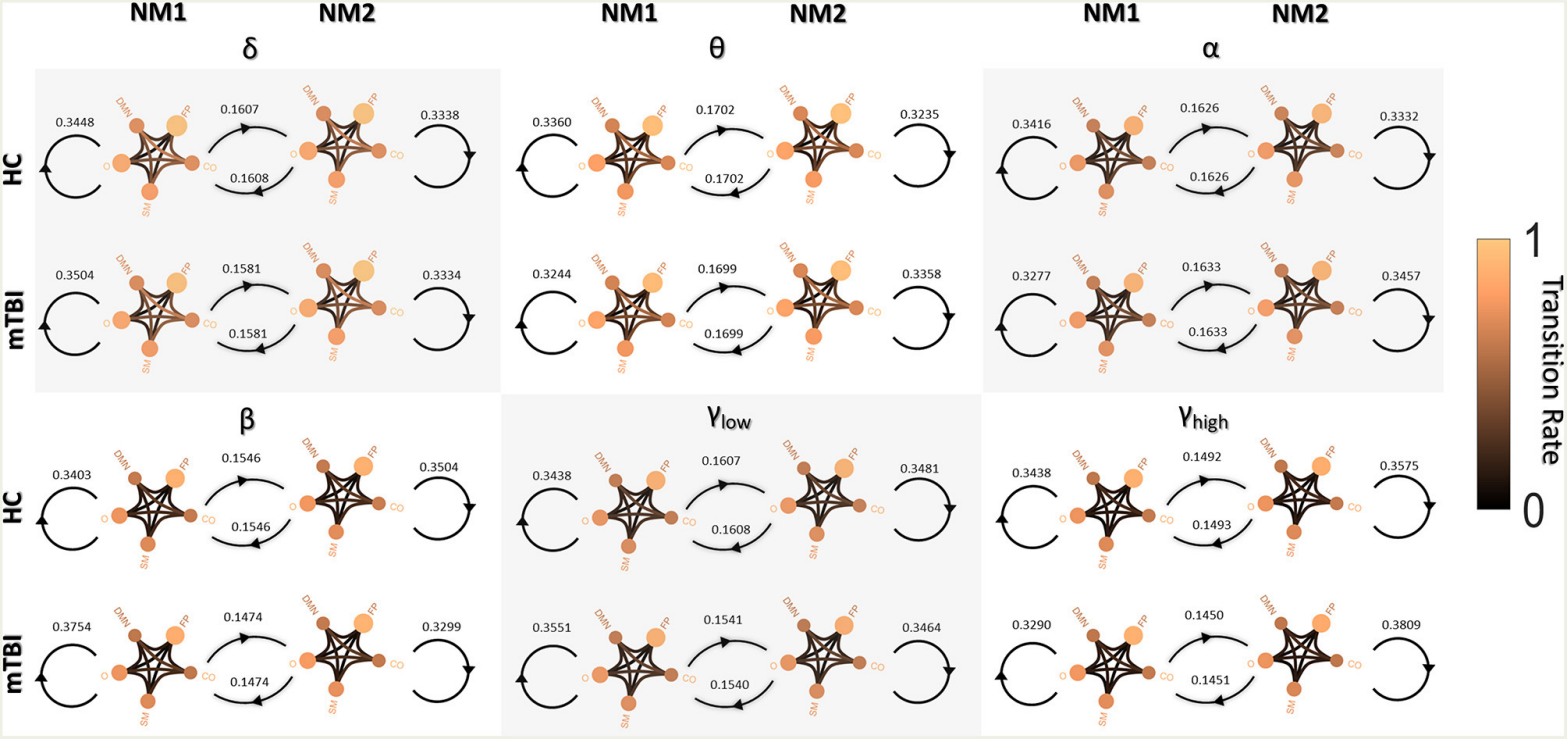
Pairwise transition diagram between NMstates for each group and frequency band. Transitions between and within network microstates are presented for every frequency band. The value in every edge denotes transition rate, with the total sum being equal to 1. The color and size of a node encode the mean degree of each sub-network, while the color of between sub-networks connection encodes the mean functional strength between the ROIs that constitute each sub-network. Both strength and degree are normalized using the maximum value between the two groups and all frequency bands. The color bar is common to all cases. Every NMstate is represented with the corresponding label NM_i_ (for *i* = 1, 2).

Figure 3 shows the transitions between two NMstates (NM1 and NM2) for all groups and frequency bands. NMstates are sketched as 5-to-5 networks, where the size and color of a node encode the mean degree within a subnetwork, whereas the color of the between sub-network connections encodes the functional strength among the ROIs that constitute those sub-networks. A stronger connection occurs between the CO and DMN compared to the other network connections (see DMN—OCC connections in every 5-to-5 network). An overall reduction in averaged strength is observed for all the network connections among the 5-to-5 networks from the lowest frequency band to the highest frequency band. The bidirectional transition rate was higher for the HC group in all the frequency bands apart from α. The self-loop transition rate for the NM1 was higher for the mTBI than the HC in the frequency bands δ, β and γ_low_ and for the HC group in the rest frequency bands. Regarding the self-loop transition rate for the NM2, higher values were observed for the HC in the frequency bands δ, β and γ_low_ and for the mTBI in the rest frequency bands.

### Aberrant Higher-Lower Mean Degree for mTBI Subjects

**Figure 4:**
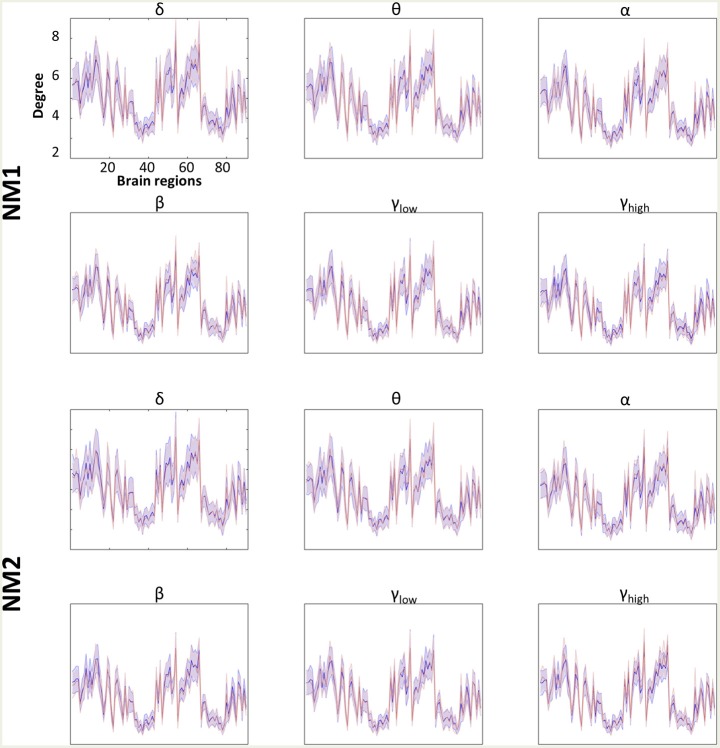
Averaged node degree per brain region. Overlapped groups (control: blue and mTBI: red) averaged node degree per NMstate (NM_i_ for *i* = 1, 2) and frequency band (δ, θ, α, β, γ_low_, γ_high_) for every brain region (horizontal axis). No statistical differences occurred after the statistical evaluation (see section Network Metrics Derived From NMstates and Brain Subnetworks).

**Figure 5:**
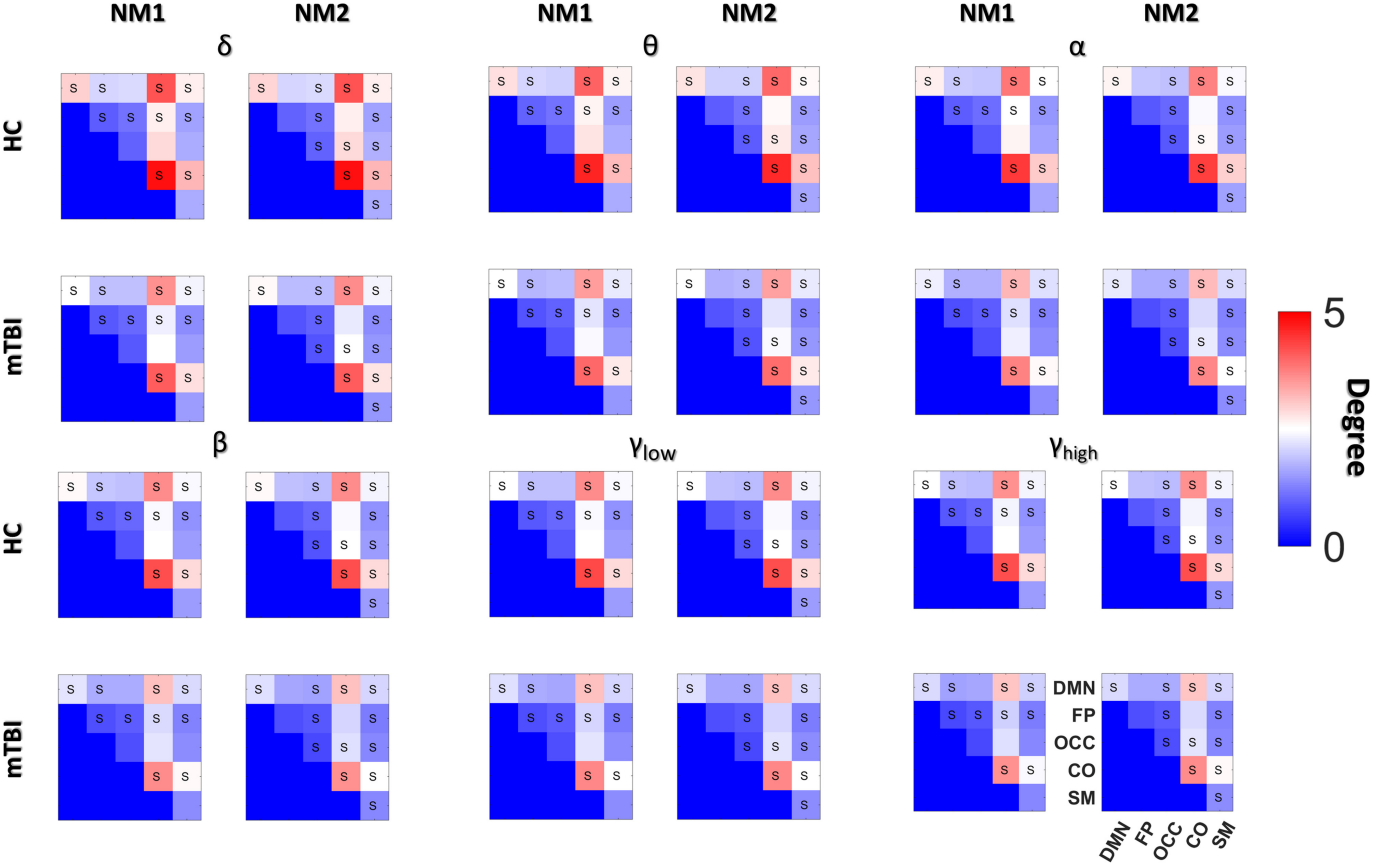
Averaged node degree with and between subnetworks. Group (HC: blue and mTBI: red) averaged node degree per NMstate (NM_i_ for *i* = 1, 2) and frequency band (δ, θ, α, β, γ_low_, γ_high_) for every basic anatomical network (BAN: DMN, default mode network; FP, frontoparietal; OCC, occipital; CO, cingulo-percular; SM, sensorymotor). The diagonal voxels depict averaged node degree within every brain subnetwork while the non-diagonal voxels represent averaged node degree between every pair of BANs. Statistical comparisons are presented between the two groups and the survived differences during the FDC adjustment are noticed using the symbol “S.” Statistical evaluation was based on the procedure described in section Network Metrics Derived From NMstates and Brain Subnetworks.

Figures 4, 5 summarize the mean degree for two NMstates (NM1 and NM2) across the frequency bands in both groups and each brain region, within and between the five subnetworks. We found statistically significant differences between the two groups in most within and between brain regions, but no differences were observed on the mean degree of the individual brain regions. A separate statistical group comparison was followed for each NMstate and frequency band. Our analysis revealed an aberrant higher-to-lower pattern of degree reduction for mTBI subjects compared to the HC group.

Then we compared the mean degree between HC and mTBI, only for the cases that survived the FDR adjustment. We started the comparison driven by NMstates and then by frequency bands and we continued with the individual brain regions, the within or between brain subnetworks. The total number of tested hypotheses (whether mTBI and HC have an equal mean degree) was 1,080 (6 bands × 2 NMstates × 90 brain regions) and 180 (6 bands × 2 NMstates × 15 BAN combinations). Before FDR correction, there were 138/1,080 and 133/180 *p*-values smaller than 0.05. No *p*-values survived for the individual brain regions but 116 (58 per NMstate)/180 *p*-values survived after FDR correction. In the next paragraph, we describe only the statistical differences occurred in the mean degree of within or between brain subnetworks cases.

For the NMstate 1 (NM1 in Figure 5), the mean degree for HCs was higher in 38 cases (14 within BAN−2 in δ up to β and 3 for the γ_low_ and 3 for the γ_high_, and 24 between BAN−6 in θ and α, 1 in δ and β, 7 in γ_high_, and 3 in γ_low_) whereas the mean degree for mTBI was higher in 20 cases (7 within BAN−3 in δ, 1 in α and γ_low_, and 2 in β, and 13 between BAN−6 in β, 4 in δ, 2 in α, and 1 in γ_low_). Furthermore, in all frequency bands, the mean degree within the CO and DMN showed always the highest degree for HC compared to mTBI group, while the mTBI group showed a higher degree within OCC and SM compared to the HC group. The degree between CO-DMN reached the highest value for the HC group compared to the mTBI group, while the mTBI group showed the highest degree in FP-DMN. With regard to the mean degree in NMstate 2 (NM2 in Figure 5), the HC degree was higher in 35 cases (15 within BAN−2 in δ up to α and γ_high_, 4 in γ_low_ and 3 in β, and 20 between BAN−2 in α and γ_low_, 5 in δ, 3 in θ, 7 in β and 1 in γ_low_) while mTBI showed higher degree values in 23 cases (6 within BAN−2 in θ and γ_high_, 1 in α and γ_low_, and 17 between BAN−2 in θ and γ_low_, 6 in α and 7 in γ_high_). In addition, we observed the same trend in NM2 for within and between BAN for the HC and mTBI groups as in NM1.

### Abnormal Fluctuations of Topological Mapping Profile in mTBI

**Figure 6:**
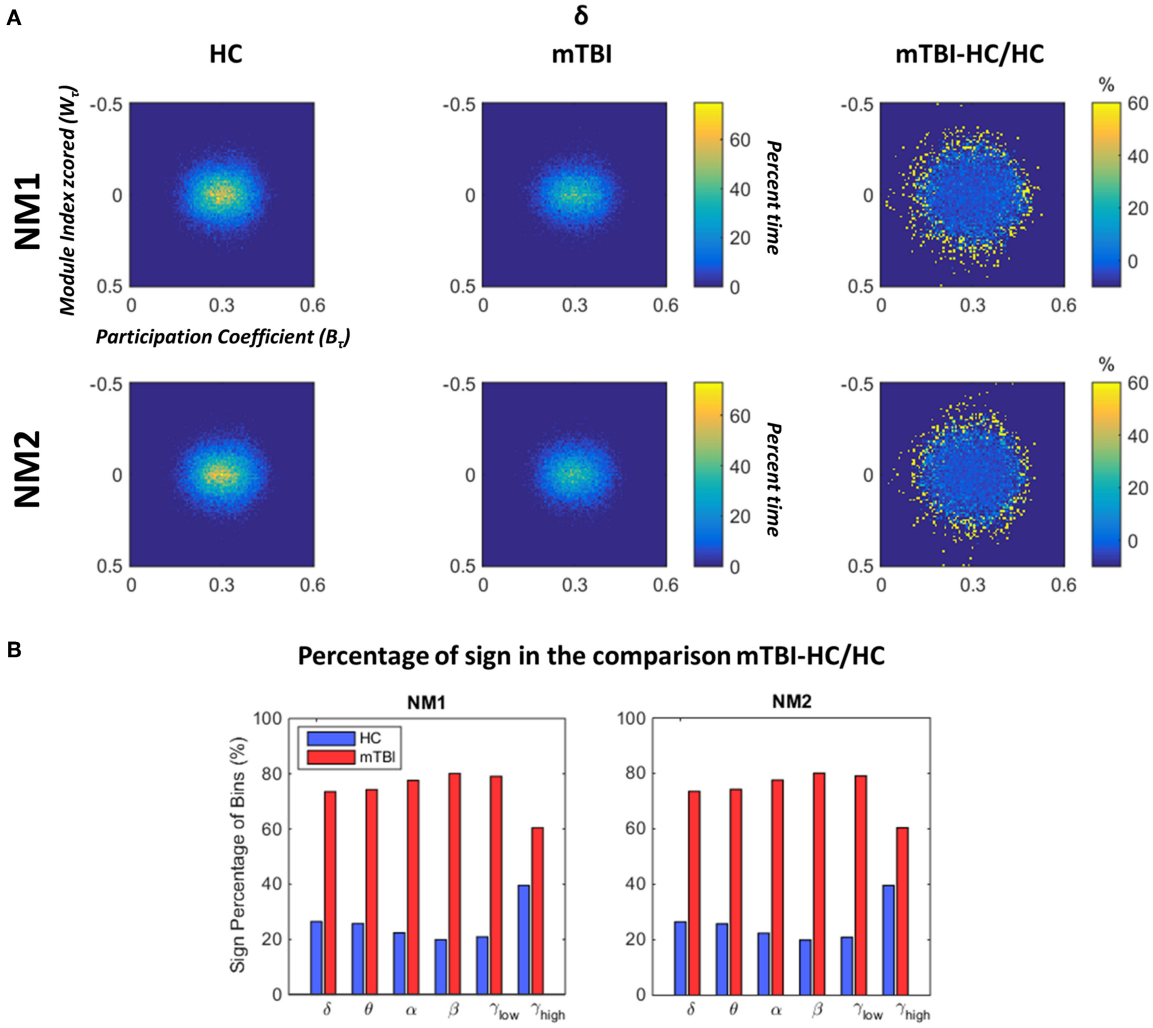
Dynamic fluctuations in topological mapping and group differences. **(A)** The topological mapping (TM) of the frequency band δ for both network microstates (upper and lower rows) for every group (HC: left column and mTBI: right column). The relative distance between the TM of the two groups is presented (TM_mTBI_ – TM_HC_/TM_HC_ in %) in the right column. The module degree Z-scored *W*_τ_ (vertical axis) and the participation coefficient *B*_τ_ (horizontal axis) are presented in the common histogram showing percent time. The color bar for the HC and mTBI groups is common, while a different color bar is used for the relative distance between the HC and the mTBI. **(B)** The sign percentage of bins is presented in this picture indicating the number of non-zero bins from the TM that appear positive (i.e., higher TM values for the mTBI) or negative (i.e., higher TM values for the HC) per frequency band and network microstate (NM_i_ for *i* = 1, 2).

In Figure 6 we depict the topological mapping (TM) for the frequency band δ (Figure 6A) and a comparison on the number of the non-zero bins is shown in its second part (Figure 6B). We present this band as an example as similar patterns were observed for the rest of the frequency bands with tiny changes (see section Results in Supplementary Material). The TM profiles were higher for the HC than mTBI groups for this specific band in both network microstates (Figure 6A, left and middle columns), while the relative difference between the two groups showed more positive values for mTBI compared to the HC, indicating a higher temporal modular stability of the HC compared to the mTBI (Figure 6A, right column). This higher entropic profile of temporal modular architecture of mTBI NMstates revealed a new feature for characterizing temporal functional brain networks. This relative difference is illustrated in Figure 6B based on the percentage of the non-zero bins of the histogram (see Figure 6A). We observed that the sign percentage of the non-bins was substantially higher for the mTBI (>70%–positive) than the HC group (<40% negative) consistent across the frequency bands and NMstates.

**Figure 7:**
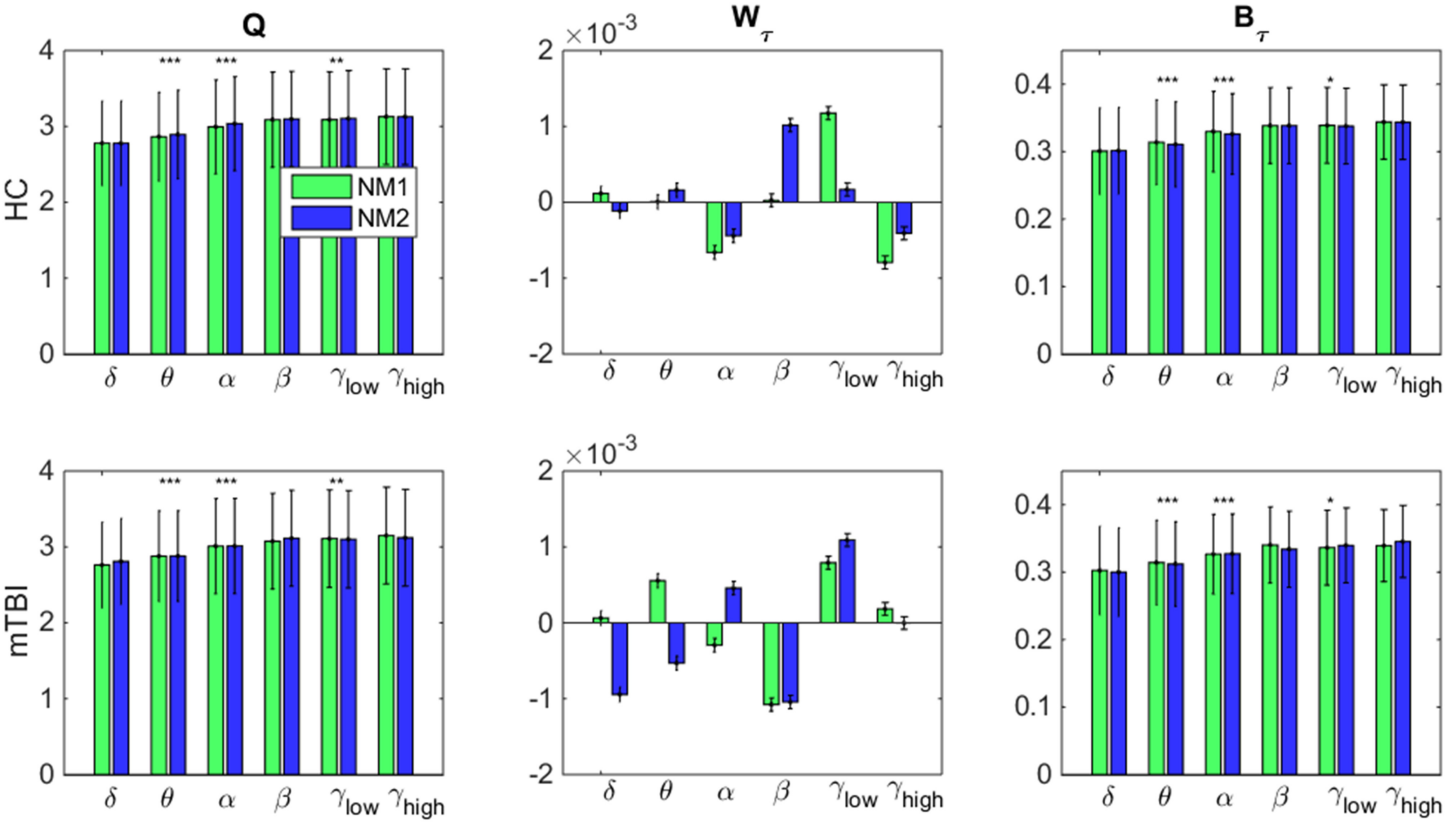
Network microstates comparison. A statistical comparison between the network microstates (NM1 in green and NM2 in blue) is presented for the modularity index (left column: **Q**), the module degree Z-scored (middle column: **W**_**τ**_) and the participation coefficient (right column: **B**_**τ**_) for both groups. The presented bar plots with error bars show the average value and standard deviation across all brain regions and subjects separately for every group (upper row: healthy control − HC, low row: mTBI). The occurred statistically significant differences are depicted based on a star-like representation (*p < 0.05, **p < 0.01, and ***p < 0.001). Statistical evaluation was based on the procedure described in section Chronnectomics: Characterization of Temporal Dynamics of NMstates.

In Figure 7, we examine whether the average value across all brain regions and subjects per group (HC: upper row and mTBI: lower row) for every network metric (*Q*: modularity index, W_τ_: module degree Z-scored and B_τ_: Participation coefficient) and frequency bands, was significantly different between the networks microstates (NM1 and NM2).

Focusing on *Q* in both groups (Figure 7, left column), higher values for NM2 than NM1 were estimated for all frequency bands apart from δ and γ_high_ and statistically significant only for the frequency bands θ, α (NM2 > NM1, *p* < 0.001) and γ_low_ (NM2 > NM1, *p* < 0.01). We observed that no significant differences occurred between the NMstates for the network metric W_τ_, even though, larger variations occurred between them across all frequency bands and groups (Figure 7, middle column). On the last column of Figure 7, the average value of B_τ_ across brain regions and subjects is found significantly higher for the NM1 than NM2 in the frequency bands θ, α (*p* < 0.001) for both groups. For γ_low_, we observed that NM1 was significantly higher that NM2 (*p* < 0.05) for HC group but, for mTBI, we observed the opposite (NM2 > NM1, *p* < 0.05).

### Classification Performance With Chronnectomics and NMstates

**Figure 8:**
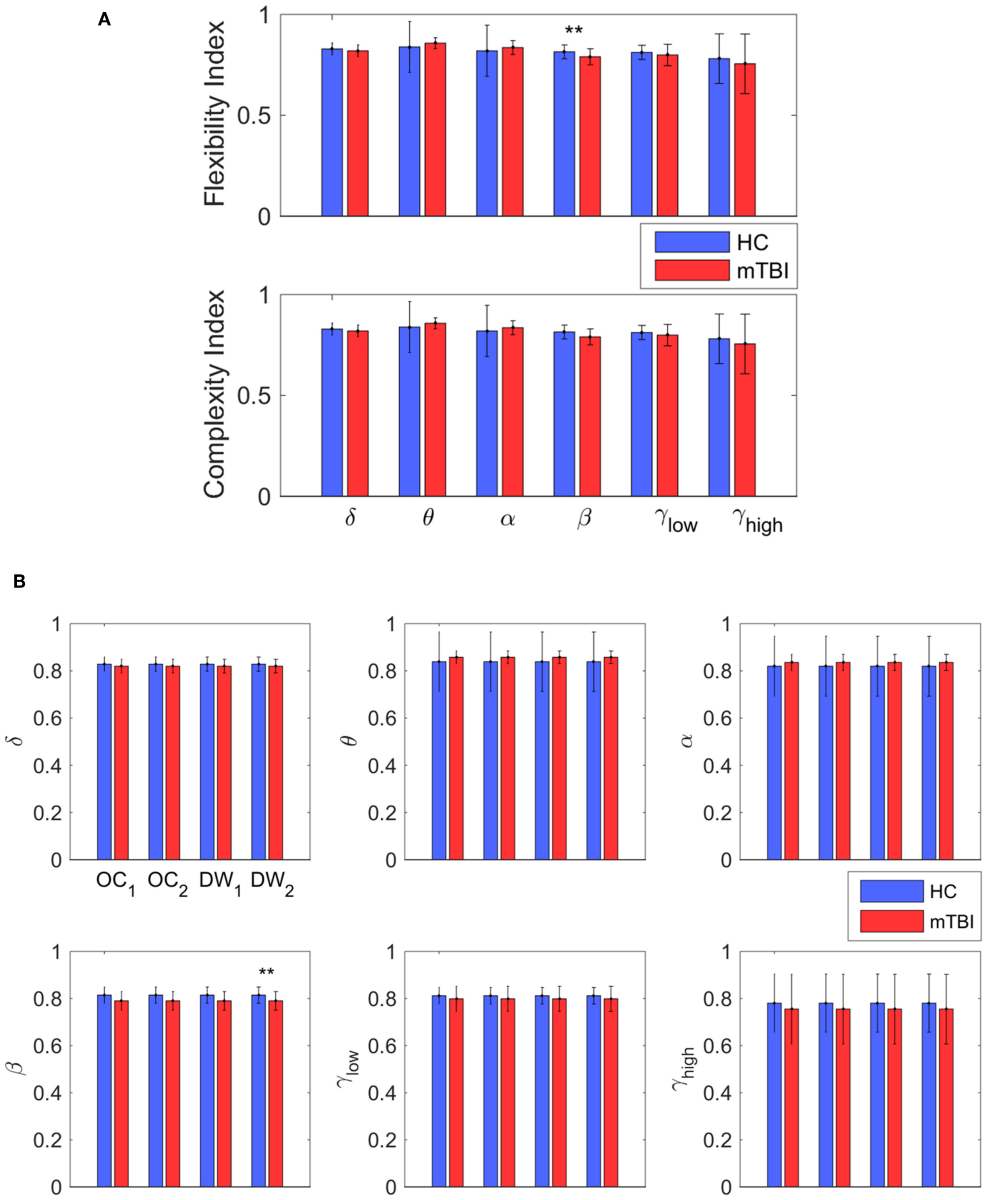
Discrimination between the two groups. **(A)** The average value and standard deviation of each metric (transition probability, complexity, occupancy time, and dwell time) and group (blue for control and red for mTBI) for every frequency band. Statistically significant difference on mean value that survive for every metric between the two groups are noticed (*p < 0.05, **p < 0.01, and ***p < 0.001) **(B)** The most informative features, as categorized by ranking analysis, that result in complete separation between the control (blue) and the mTBI (red) groups. Statistical evaluation was based on the procedure described in section Chronnectomics: Characterization of Temporal Dynamics of NMstates.

Table 2 summarizes the results of classification performance (in % for accuracy, sensitivity, and specificity) based on two NMstates (NM1 or NM2) and all chronnectomic features in all the frequency bands. With regard to the ability of the NMstates to discriminate the two groups (Table 2A), high classification performance was observed for both NMstates. In particular, the highest accuracy (>96%) was observed in the α, β, and γ_low_ frequency bands for NM1, while the rest of the cases also showed very high accuracy (>91%). The sensitivity was always 100% but the specificity ranged from 77 to 87%. Table 2B summarizes the classification results obtained using all chronnectomics metrics, namely Flexibility Index (FI), Occupancy Time (OT), and Dwell Time (DW), and the Complexity Index (CI). As can be seen, classification performance was much lower (80%) than in the previous case, showing good sensitivity but very low specificity. Figure 8A shows the group average FI and CI features for every frequency band, whereas Figure 8B depicts OT and DW per NMstate and frequency band. Statistical analysis revealed significant higher FI and DW in NM1 for the HC group compared to the mTBI group in the β frequency band.

**Table 2:** Summary of the classification performance (Accuracy, Sensitivity, and Specificity in %) **(A)** per frequency band for every NMstate NMstate (NM1 or NM2), and **(B)** including all chronnectomics (Flexibility Index, Occupancy Time, and Dwell Time) and Complexity Index (CI) for all the frequency bands.

**NMstates**	**Frequency band**	**Accuracy (%)**	**Sensitivity (%)**	**Specificity (%)**
**A**				
NM1	δ	91.27 ± 1.34	100	77.30 ± 3.49
	θ	92.27 ± 1.35	100	80.00 ± 3.49
	α	96.14 ± 0.55	100	90.00 ± 1.42
	β	97.42 ± 0.67	100	93.33 ± 1.74
	γ_low_	93.85 ± 1.22	100	83.97 ± 3.18
	γ_high_	96.15 ± 0.65	100	90.00 ± 1.68
NM2	δ	93.56 ± 0.73	100	83.33 ± 1.87
	θ	94.85 ± 0.69	100	86.67 ± 1.83
	α	93.84 ± 0.70	100	83.97 ± 1.88
	β	92.88 ± 0.98	100	81.40 ± 2.53
	γ_low_	93.85 ± 1.22	100	83.97 ± 3.18
	γ_high_	95.16 ± 0.54	100	87.40 ± 1.39
**B**				
Chronnectomics + CI		80.34 ± 1.34	99.65 ± 0.32	49.23 ± 3.56

## Discussion

In the current study, we developed a framework for analyzing the spatiotemporal evolution of functional connectivity patterns of the MEG source-reconstructed activity at rest for mTBI and HC subjects. Each frequency-dependent DFCG was discretized via the neural-gas algorithm into a symbolic time series that described the temporal evolution of brain states (NMstates). DFCGs were treated as first order Markovian chains from which valuable chronnectomic markers were estimated. Our results revealed significantly lower values of the flexibility index (FI) and dwell time (only for the second NMstate) for the mTBI subjects compared to the HC subjects in the beta frequency band. Following a machine learning approach, we were able to discriminate the two groups with an 80% accuracy using the chronnectomics derived from the whole set of frequency bands. In contrast, we obtained a higher classification performance using two NMstates (vectors of 90 eigenvalues) reaching 94% average accuracy across all frequency bands. The estimated topological mapping profiles (module degree W_T_ vs. Participation Coefficient B_T_), summarizing time-resolved modularity organization of the NMstates, revealed a higher entropy for the mTBI group compared to healthy controls (Figure 6), consistently across frequencies and NMstates. This topological mapping profile was spatially concentrated for the HC group, while for mTBI was dispersed across the 2D map and this behavior was quantified by the percentage of positive—negative group difference shown in Figure 6B. The proposed analysis procedure proved valuable for characterizing the altered whole-brain transitions of characteristic NMstates in mTBI patients compared to controls.

It is strongly believed that long distance functional connections are mainly maintained between brain areas that communicate in low frequency bands (delta and theta), while local or more short-distant functional connections are mainly observed between brain areas oscillating in the beta and gamma frequencies (von Stein et al., 1999; Thatcher et al., 2008). Specifically, in mTBI studies, Sponheim et al. (2011) reported reduced brain connectivity between specific frontal electrode positions for delta, beta, and gamma frequency bands while Thornton (2003) adopted an audio memory task paradigm and reported that phase and coherence were lowered for the beta1 and beta2 spectral bands (for a review see Rapp et al., 2015). In a follow-up study, they reported an increased connectivity pattern for higher frequencies including alpha, beta frequency and a decreased connectivity pattern for delta, theta brain frequencies (Castellanos et al., 2010). Human beta oscillations (13–30 Hz) are mainly associated with sensorimotor processing (Symons et al., 2016). However, they have been recently linked to attention, emotion, and cognitive control (Guntekin et al., 2013; Symons et al., 2016). Beta frequency is also related to active thinking, focus, high alertness and anxiousness; it is the dominant rhythm in alert or anxious patients.

A recent study in animals and humans, with the support of a biophysical model, tested the theory that beta frequency decrease functions related to sensory or motor information processing in the whole brain (Sherman et al., 2016). They proved that beta is not a byproduct of brain activity but beta signals rather come from the thalamus.

Beta expression and its coherence between distinct brain foci are thought to contribute to information processing at several levels, including communication between neocortical areas (Bressler and Richter, 2015). Sherman's study provided a unifying link between studies suggesting that beta coordination mediates top-down neocortical processing (Engel and Fries, 2010) and studies showing that top-down influences are communicated through supragranular layers (Rockland and Pandya, 1979). Taking all together, these studies and others suggest that beta frequencies inhibit information processing and decrease focally to allow optimal information relay.

The previous results confirmed three predicted hypotheses—mechanisms where neocortical beta burst events would possibly decrease information relay. The first states that the inputs creating beta may stimulate inhibitory neurons in the top layers of the cortex. The second is devoted to a possible saturation of the activity of pyramidal neurons which, as a consequence, reduce their ability to process information. The third argues that the thalamic bursts producing beta cover the majority of the thalamus, so that it cannot pass information to the cortex. In close analogy to neocortical beta states is the decreased relay of bottom-up motor or sensory information via the thalamus during thalamic alpha states. Possible mechanisms that support this sensory relay during alpha frequency are the following: thalamic hyperpolarization, synaptic depression at thalamocortical synapses, and low capacity for novel external information relay during the co-occurrence of internally generated alpha rhythms in conjunction to beta states (Klimesch, 2012). Our findings in beta frequency with internal co-occurrence of alpha states in relation to the aforementioned hypotheses support a central role of the thalamus impairment in mTBI (Grossman and Inglese, 2016). Sensorimotor driven paradigms applied to mTBI subjects could shed light and further enhance the interpretation of our findings and Sherman's hypotheses for the mechanisms producing neocortical beta rhythms.

Recent advances in MEG and network neuroscience have shown that mTBI can be manifested as an excessive pattern of slow-wave activity (Huang et al., 2012), while localization of this slow-wave activity can reveal the foci of the damage (Huang et al., 2014). Dimitriadis et al. (2015) employed PLV to quantify time-static FCGs at the sensor level. They revealed a dense local and sparse long-range connectivity pattern for healthy controls and a sparse local and dense long-range pattern for the mTBI subjects. Activity in the alpha frequency band was the most discriminative feature for separating the two groups. In our previous studies (Antonakakis et al., 2015, 2016, 2017a), we combined intra- and inter- frequency couplings in a single FCG, showing a dense network of stronger local and global connections for HC group, in agreement with other studies (Rapp et al., 2015). In our most recent study (Antonakakis et al., 2017b), the mTBI group showed hyper-synchronization in a rich-club network organization compared to the HC group.

Structural neuroimaging combined with diffusion tensor imaging (DTI) has revealed an association of mTBI with aberrant white matter microstructures (Huang et al., 2009). Specifically, a connection was shown between focal increased slow wave activity and the location of white matter injury, which was consistent with the hypothesis that the deceleration of oscillations could be caused by differentiation (Llinás et al., 1999).

A disrupted inter-regional frequency-dependent functional connectivity pattern has also been reported in combat-related blast injury using EEG (Sponheim et al., 2011). Oscillatory functional synchronization between brain areas play a pivotal role in network connectivity and support both cognition and behavior (Ward, 2003; Uhlhaas et al., 2009). The expression of this network connectivity in various frequencies at resting-state is related to the intrinsic multi-frequency organization of brain activity that is pertinent to healthy brain function and dysfunction in various clinical populations (Engel et al., 2014).

The present study explored for the very first time the consequences of mTBI in human brain functionality at resting-state using MEG and DFCG analysis. Our analysis summarized DFCGs with connectivity graphs, where each graph was associated with one NMstate (Functional Connectivity microstate or alternatively brain state). The major outcome of this analysis was a symbolic time series for the temporal description of the evolution of these brain states. Dynamic functional connectivity graphs were treated as Markovian chains from which a variety of chronnectomic metrics were extracted. Recent studies defined microstates in MEG and fMRI modalities using a Hidden Markovian Modeling (HMM) approach employing the band-limited amplitude envelope of virtual source time series (Baker et al., 2014) and the BOLD time series, respectively (Vidaurre et al., 2016). Both methodologies defined microstates in a full analogy to original EEG microstates, via a mining procedure of brain activity rather than the dynamic functional connectivity. Machine learning analysis over the derived chronnectomics produced a high classification accuracy (94%) between healthy controls and mTBI subjects using the NMstates, and an 80% performance when employing chronnectomics across frequencies (Table 2). Compared to the 92% accuracy reported by other studies (Vergara et al., 2018,), we reached a slightly higher discrimination between to the two groups benefiting from the dynamic content of the network analysis.

Functional MRI (fMRI) studies at resting-state have revealed numerous discrete functional neural networks (De Luca et al., 2005; Damoiseaux et al., 2006). These neural networks include well-described motor, sensory, language, and visual networks (Cordes et al., 2000). In addition, the set of characteristic neural networks comprise brain areas that engage various higher-order cognitive operations that are impaired in TBI-like FP and CO brain subnetworks (Beckmann et al., 2005; Dosenbach et al., 2007; Seeley et al., 2007). The vast majority of the resting brain's resources (~80%) is expended to maintain the homeostasis between the resting-state networks (Raichle and Mintun, 2006). This observation suggests that resting-state networks are excellent indicators of disruptions of coordinated functional connectivity between brain areas in many disorders and diseases, including mTBI. Researchers have shown a particular interest in the “default mode” network, which is a group of interconnected midline cortical regions that show high activity in the absence of externally-imposed cognitive processing (Buckner et al., 2008). The DMN reduces its activity during cognitive demands and may be involved in self-referential processing and internal emotional states (Power et al., 2011; Barch, 2013). Finally, CO plays a central role in sustaining alertness and attention (Coste and Kleinschmidt, 2016).

Figure 5 demonstrates the global mean degree of the within or between brain region subnetworks for every NMstate across frequency bands in both groups. Our statistical analysis applied independently to every NMstate revealed significant differences between the two groups in most cases. Our analysis revealed an aberrant higher-lower pattern for mTBI subjects compared to healthy controls. The central brain networks that were more connected with the rest of the brain were the CO and DMN across all frequency bands. In mTBI patients, DMN and CO were less connected with the rest of brain networks compared to healthy controls while the degree between CO-DMN was the highest for HC compared to the mTBI while mTBI group showed the highest degree in FP-DMN across all frequency bands (Figure 5). Our results provided the interesting finding that CO and DMN were more central hubs in HC compared to mTBI while CO-DMN were better interconnected also in HC. These findings revealed that attention, sustaining alertness, and other cognitive functions like thinking of others or themselves, remembering the past and planning of the future, that are linked to CO and DMN (Buckner et al., 2008) are impaired in mTBI. This is the very first study that revealed such interesting findings, linking the consequences of mTBI with neuromagnetic whole-brain resting-state networks under the notion of dynamic functional connectivity on the source level. Furthermore, our analysis demonstrated a high classification performance which further enhanced the significance of the proposed procedure. Our study employed beamformed source-reconstruction of resting-state MEG activity in various frequency bands with a common MRI template for all subjects. However, a recent study explored the deviation in power and connectivity of virtual source MEG activity when using a template instead of native MRIs (Douw et al., 2017) and found that relative power, connectivity measures, and network estimates were consistent in both cases.

Figure 6 showed that the time-resolved FCD of the mTBI group are more randomly organized in time compared to the HC group. This is a major result indicating that the specific brain insult may lead to an uncertain network organization over time. However, the present result needs further validation from different mTBI cohorts in order to confirm the temporal instability of the time-resolved network alterations.

With regard to the limitations of the present study, a possible criticism might be the absence of individual MRI data, which potentially could result in more precise source reconstructions. Furthermore, no clinical scores were available for the mTBI group and, therefore, a statistical comparison with the results of the present study was not feasible. Finally, it is worth mentioning that the connectivity metric used did not consider directionality, i.e., the direction of information flow between two interconnected nodes, and the interdependency between frequency bands, as it was presented in our previous study (Antonakakis et al., 2017b). For this purpose, we will conduct a future study to alleviate this limitation.

## Conclusions

In the present study, we examined for the very first time how mTBI affects the dynamics of functional brain networks on beamformed source-reconstructed resting-state activity. Dynamic functional graphs were treated as Markovian chains via a well-established analytic framework that discretized their temporal evolution into a symbolic time series. Symbolic dynamics and chronnectomics have already proven valuable in the discrimination of healthy controls from mTBI subjects. We further propose that network microstates (NMstates) form a valuable connectomic biomarker for detecting mTBI succeeding an average performance of 94% across frequencies. Cingulo-Opecular (CO) and Default Mode Network (DMN) were the central network hubs of the derived brain states (NMstates), where mTBI subjects were less connected with the rest of the brain networks and showed substantially smaller time-resolved organization compared to healthy controls. mTBI subjects showed a higher entropic temporal evolution of modular organization compared to healthy controls. A significant difference on the flexibility index was observed between the two groups for the β frequency band which may support a central role of the thalamus impairment in mTBI. Finally, in a future study, it would be interesting to evaluate the sensitivity of the current analysis approach and chronnectomic features in detecting the return of mTBI subjects back to normal.

## Data Availability Statement

The datasets generated for this study will not be made publicly available in accordance with the consent form signed by the subjects. However, requests to see the raw data can be sent to the corresponding author.

## Ethics Statement

The studies involving human participants were reviewed and approved by the Institutional Review Board (IRB) and the Human Research Protection Official Review, Department of Defense. The patients/participants provided their written informed consent to participate in this study.

## Author Contributions

SD conceptualized the research analysis with regard to source connectivity, the connectivity metrics, and the classification scheme. MA contributed to the design of data preprocessing, the statistical assessments, and performed all analyses. In collaboration with SD, MA prepared the figures and drafted the manuscript. AP designed the original study and GZ contributed to the collection of the data and preprocessing methods. MZ, AP, and GZ provided the input on the development of and critique on the revisions of the manuscript. All authors read and approved the final version of the manuscript.

### Conflict of Interest

The authors declare that the research was conducted in the absence of any commercial or financial relationships that could be construed as a potential conflict of interest.

## Footnotes

^1^https://github.com/stdimitr/topological_filtering_networks.

^2^http://www.brain-connectivity-toolbox.net/.
