# Supplementary material for: Aberrant Whole-Brain Transitions and Dynamics of Spontaneous Network Microstates in Mild Traumatic Brain Injury
Source: Front Comput Neurosci. 2020 Jan 15;13:90. doi: 10.3389/fncom.2019.00090 (PMC6974679; doi:10.3389/fncom.2019.00090)
Supplement: Supplementary file 1 [file Presentation_1.zip › Supplementary Materials.DOCX]

Supplementary Material

# Aberrant Whole-Brain Transitions and Dynamics of Spontaneous Network Microstates in Mild Traumatic Brain Injury

Marios Antonakakis^1,2*^, Stavros I. Dimitriadis^3,4,5,6,7^, Michalis Zervakis^8^, Andrew C. Papanicolaou^9^, and George Zouridakis^10^

1. Institute for Biomagnetism and Biosignal Analysis, University of Muenster, Muenster, Germany,
2. Neuroinformatics Group, Cardiff University Brain Research Imaging Center (CUBRIC), School of Psychology, Cardiff University, Cardiff, United Kingdom,
3. Institute of Psychological Medicine and Clinical Neurosciences, Cardiff University School of Medicine, Cardiff, United Kingdom,
4. Cardiff University Brain Research Imaging Center (CUBRIC), School of Psychology, Cardiff University, Cardiff, United Kingdom,
5. School of Psychology, Cardiff University, Cardiff, United Kingdom,
6. Neuroscience and Mental Health Research Institute, Cardiff University, Cardiff, United Kingdom
7. MRC Centre for Neuropsychiatric Genetics and Genomics, School of Medicine, Cardiff University, Cardiff, United Kingdom
8. Digital Image and Signal Processing Laboratory, School of Electronic and Computer Engineering, Technical University of Crete, Chania, Greece,
9. Departments of Pediatrics, and Anatomy and Neurobiology, Neuroscience Institute, University of Tennessee Health Science Center, Le Bonheur Children’s Hospital, Memphis, TN, United States,
10. Biomedical Imaging Lab, Departments of Engineering Technology, Computer Science, Biomedical Engineering, and Electrical and Computer Engineering, University of Houston, Houston, TX, United States.

***Corresponding author**

Marios Antonakakis

Ph.D. Student

Institute of Biomagnetism and Biosignal Analysis

Westfalian Wilhelms-University Muenster

Malmedyweg 15, 48149 Muenster, Germany

Tel.: +49-251-83-52547

Email: [marios.antonakakis@uni-muenster.de](mailto:marios.antonakakis@uni-muenster.de), [antonakakismar@gmail.com](mailto:antonakakismar@gmail.com)

# Methods

## The Neural Gas Algorithm

Neural gas is an artificial neural network based on the self-organizing map^[[1]](#footnote-1)^ and introduced in 1993 by Martinetz and colleagues (Martinetz et al., 1993). Neural gas is an optimization algorithm for finding data representations based on feature vectors. The algorithm was coined "neural gas" because of the dynamics of the feature vectors during the adaptation process, which distribute themselves like a gas within the data space. It is applied where data compression or vector quantization is an issue, for example speech recognition (Angelopoulou et al., 2005), image processing (Curatelli, Mayora-Iberra, 2000), electrophysiological signal processing for coding temporal brain dynamics (Dimitriadis et al., 2010).

Given a probability distribution *P(v)* of data vectors *v of the 2D matrix V* (see definition in subsection 2.4.2, main text) and a finite number of feature vectors *f_i_, i=1,...,N*. With each timestamp *ts* a data vector randomly chosen from *P* is presented. Subsequently, the distance order of the feature vectors to the given data vector *v* is determined. The term *i_0_* denotes the index of the closest feature vector, *i_1_* the index of the second-closest feature vector, etc. and *i_N-1_* the index of the feature vector most distant to *v*. Each feature vector (*k=0,...,N-1*) is adapted then according to

$$w_{ik}^{ts+1}=w_{ik}^{ts}+\varepsilon\cdot e^{-\frac{k}{\lambda}}\cdot(v-w_{ik}^{ts})$$

where $\varepsilon$ denotes the adaptation step size and $\lambda$ is the so-called *neighborhood range*. The terms $\varepsilon$ and $\lambda$ are reduced with increasing *ts*. After sufficiently many adaptation iterations the feature vectors cover the data space with minimum representation error (Martinetz et al., 1993). The term $\varepsilon$ of the NG can be interpreted as gradient descent on a cost function. By adapting not only the closest feature vector but all of them with an iteration size decreasing with increasing distance order, compared to k-means clustering, a much more robust convergence of the algorithm can be achieved. The neural gas model retains the separates spaces without deleting nodes or creating new nodes.

# Results

In figure 1, we presented the cartography profiles for the frequency bands θ, α, β, γ_low_, γ_high_. The same cartography profile appeared for each frequency band and group (HC and mTBI). Among all these frequency bands the mTBI group appeared higher participation time than the HC group in the cartography profile representation. These relative differences (mTBI-HC/HC) indicate higher entropic profiles for the mTBI than the HC group.

| **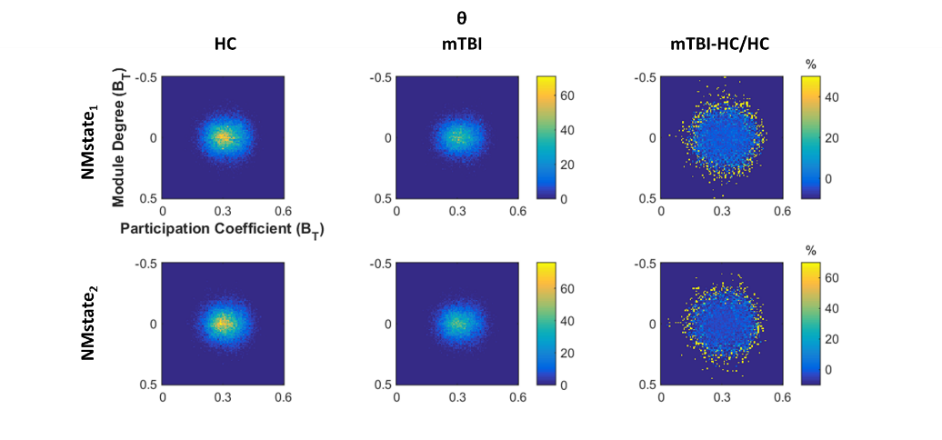** | **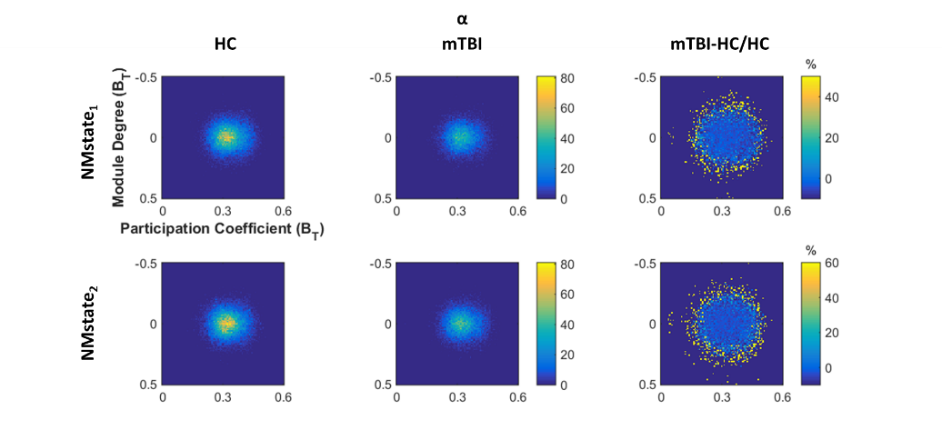** |
| --- | --- |
| **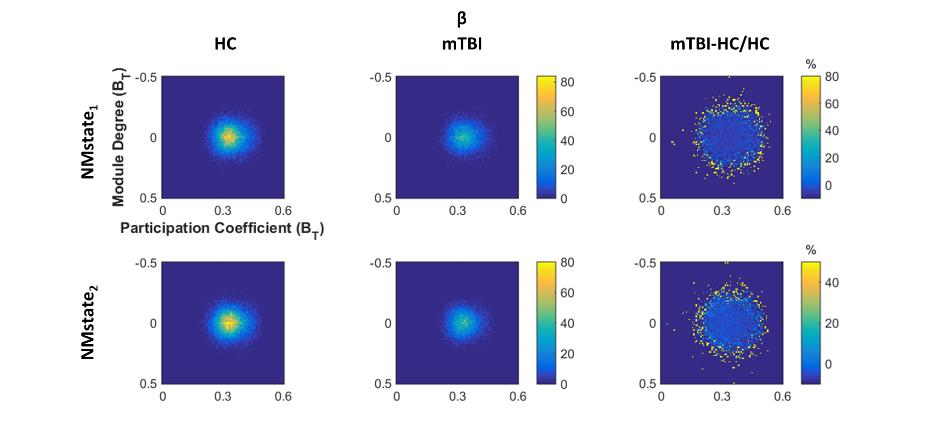** | **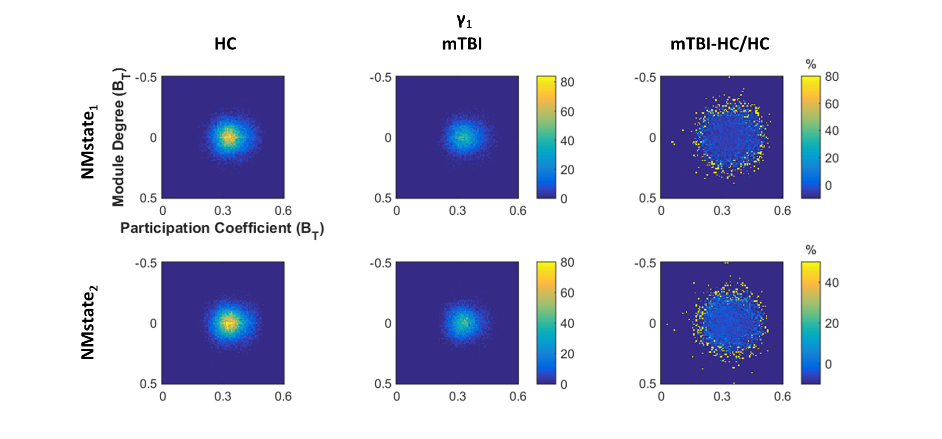** |
| **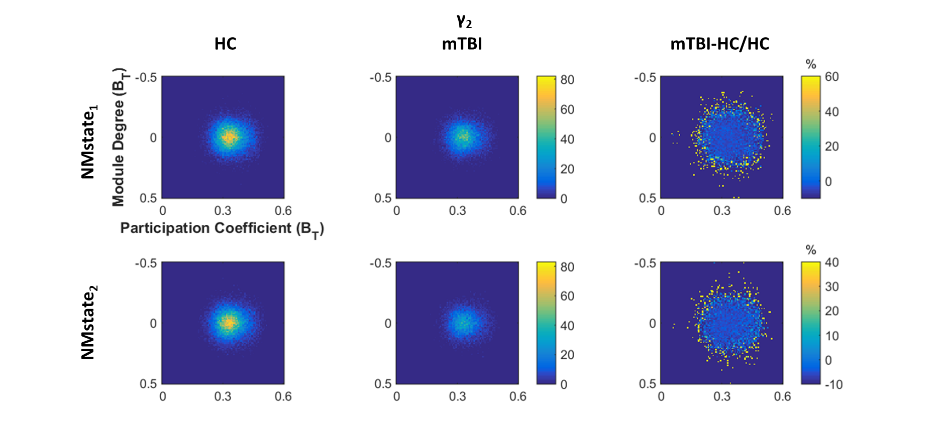** | **Figure 1. Dynamic fluctuations in cartography and group differences.** The cartographic profile (CP) of every frequency band (from the top to the bottom: θ, α, β, γ_low_, γ_high_ for both network microstates (upper and lower rows) for every group (HC: left column and mTBI: right column). The relative distance between the CP of the two groups is presented (CP_mTBI_ - CP_HC_/CP_HC_ in %) in the right column. The module degree Z-scored *W_τ_* (vertical axis) and the participation coefficient *B_τ_* (horizontal axis) are presented in the common histogram showing percent time. The colorbar for the HC and mTBI group is common, while a different colorbar is used for the relative distance between the HC and the mTBI. |

# References

Martinetz, T.M., Berkovich, S. G., and Schulten, K. J., 1993. “Neural-gas” network for vector quantization and its application to time-series prediction. IEEE Trans. Neural Netw. 4, 558–569. doi: 10.1109/72.238311

Angelopoulou A, Psarrou A, Garcia RJ, Revett, K (2005) Automatic landmarking of 2D medical shapes using the growing neural gas network. In Yanxi Liu, Tianzi Jiang, Changshui Zhang. Computer vision for biomedical image applications: first international workshop, CVBIA 2005, Beijing, China, October 21, 2005 : proceedings. Springer. p. 210. DOI:10.1007/11569541_22. ISBN 978-3-540-29411-5.

Dimitriadis SI, Laskaris NA, Tsirka V, Vourkas M, Micheloyannis S, Fotopoulos S (2010) Tracking brain dynamics via time-dependent network analysis. Journal of Neuroscience Methods 193(1):145-155.

1. <https://en.wikipedia.org/wiki/Self-organizing_map> [↑](#footnote-ref-1)
